# Exposure to aristolochic acid I compromises the maturational competency of porcine oocytes via oxidative stress-induced DNA damage

**DOI:** 10.18632/aging.101911

**Published:** 2019-04-19

**Authors:** Yu Zhang, Xiayan ShiYang, Yuwei Zhang, Yu Li, Xiaoyan Shi, Bo Xiong

**Affiliations:** 1 College of Animal Science and Technology, Nanjing Agricultural University, Nanjing 210095, China; 2 Research Center of Combine Traditional Chinese and Western Medicine, Affiliated Traditional Medicine Hospital, Southwest Medical University, Luzhou, Sichuan 646000, China

**Keywords:** Aristolochic acid, oocyte maturation, fertilization capacity, oxidative stress, DNA damage

## Abstract

Aristolochic acid (AA) is a class of carcinogenic and nephrotoxic nitrophenanthrene carboxylic acids naturally found in Aristolochia plants. These plants have been widely used as herbal medicines and also enter the human food chain as the persistent soil pollutants. It has been known that AA exposure is implicated in multiple cancer types, kidney failure and ovarian dysfunction. However, whether AA exposure would influence the oocyte quality has not yet determined. Here, we document that AAI has the negative effects on the competency of oocyte maturation and fertilization. We show that AAI exposure leads to the oocyte meiotic failure via impairing the meiotic apparatus, displaying a prominently defective spindle assembly, actin dynamics and mitochondrial integrity. AAI exposure also causes the abnormal distribution of cortical granules and ovastacin, which is consistent with the observation that fewer sperm bound to the zona pellucida surrounding the unfertilized AAI-exposed eggs, contributing to the fertilization failure. In addition, AAI exposure induces the increased levels of ROS, DNA damage and early apoptosis in porcine oocytes. Collectively, we demonstrate that AAI exposure perturbs the oocyte meiotic progression and fertilization capacity via disruption of both nuclear maturation and cytoplasmic maturation of oocyte, which might be caused by the excessive oxidative stress-induced DNA damage and apoptosis.

## Introduction

Aristolochic acids (AA), a group of active natural compounds from various herbal plants of genus Aristolochia and Asarum, have been used for traditional medicinal remedies throughout the world since antiquity and remain in use today [1,2]. The major components of AA are 8-methoxy-6-nitro phenanthro-(3,4-d)-1,3-dioxolo-5-carboxylic acid (AAI) and 6-nitro-phenanthro(3,4-d)-1,3-dioxolo-5-carboxylic acid (AAII) [3]. Both AAI and AAII are mutagenic and genotoxic, and are associated with carcinogenesis and nephrotoxicity evidenced in many animal models [4,5]. In 2012, AA was classified as carcinogenic to humans (Group 1) by the International Agency for Research on Cancer (IARC) acting by a genotoxic mechanism. Recent studies have indicated that AA exposure is linked to the upper urothelial cancers, renal cell carcinoma (RCC), liver premalignant alterations and kidney failure [6,7]. AA is also considered as the major cause of another chronic renal disease associated with urothelial malignancy named Balkan endemic nephrophathy (BEN) [8–10].

Because of these known toxicities, herbs containing AA have been restricted or banned in many countries, but it is still available in some countries in Asia and elsewhere [11]. In addition, recent studies have reported that AA enters the human food chain and causes kidney disease as a persistent soil pollutant which is taken up from the soil and bio-accumulated in food crops in a time and dose dependent manner [12]. Notably, AA is resistant to the microbial activities of soil and is highly persistent to the metabolism of plant cells, thus existing in the food products for an extended period of time [7].

The mutagenetic and carcinogenic effects of AA on humans and experimental animals are generally believed to be triggered by the formation of AA-DNA adducts, eventually leading to the accumulation of DNA damage and potentially the development of cancers and other diseases [13]. Additionally, studies have shown that AA-mediated toxicity could induce the oxidative stress in human kidney proximal tubular cells to suppress DNA repair and trigger the oxidative DNA damage [14].

Investigations of AA exposure on the ovarian function have also identified this chemical as a potent ovotoxicant. It has been reported that the ovary size and weight are significantly reduced and the number of ovulated oocytes is markedly suppressed in AAI-treated mice [15]. AAI produces strong toxic effects during ovarian development by inhibiting Akt which induces the apoptosis, suggesting that high-dose and long-term AAI treatment increases the likelihood of reproductive toxicity in women [15]. However, the molecular mechanisms regarding how AA influences the competency of oocyte maturation and fertilization has not yet determined.

In the present study, we used porcine oocytes as a research model to investigate the effects of AAI exposure on the meiotic progression and fertilization potential, because the physiological and developmental indexes of porcine oocytes such as the size of oocytes and maturation time are more similar to humans compared to mice [16]. We examined the effects of AAI exposure on the meiotic maturation in porcine oocytes through evaluating the cytoskeleton organization, mitochondrial integrity and cortical granule distribution. We also studied the influence of AAI exposure on the fertilization potential of porcine oocytes via assessing the dynamics of cortical granules and their component ovastacin as well as the sperm binding ability.

## RESULTS

### AAI exposure perturbs the meiotic maturation of porcine oocytes

**Figure 1:**
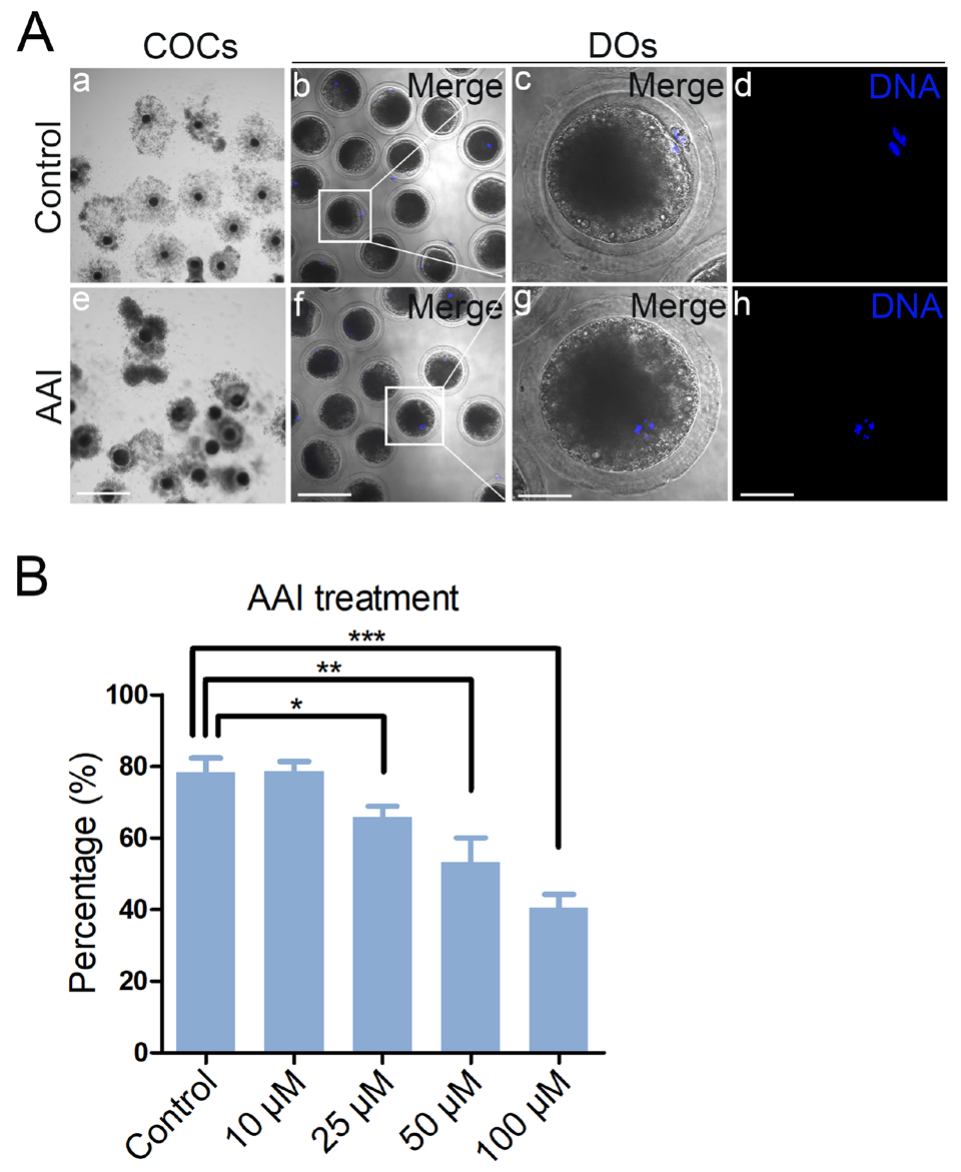
**Effects of different doses of AAI on the porcine oocyte maturation.** (**A**) Representative images of oocyte meiotic progression in control and AAI-exposed oocytes. Cumulus cell expansion of cumulus oocyte complexes (COCs) and polar body extrusion (PBE) of denuded oocytes (DOs) were imaged by the confocal microscope. Scale bar, 360 μm (a, e); 120 μm (b, f); 40 μm (c, g); 40 μm (d, h). (**B**) The rate of PBE was recorded in control and different concentrations of AAI-exposed groups (10 μM, 25 μM, 50 μM and 100 μM) after culture for 44 h *in vitro*. Data were presented as mean percentage (mean ± SEM) of at least three independent experiments. *P < 0.05, **P < 0.01, ***P < 0.001.

To test the impact of AAI on the porcine oocyte meiotic progression, increasing concentrations of AAI (10, 25, 50 or 100 μM) were supplemented to the culture medium to observe the polar body extrusion, respectively. As shown in Fig. 1A, AAI exposure prominently resulted in the oocyte meiotic arrest by displaying the poor expansion of cumulus cells surrounding COCs and the decreased number of oocytes that reached MII stage. Quantitative analysis revealed that treatment with different doses of AAI (25, 50 or 100 μM) caused a decrease of polar body extrusion (PBE) in varying degree in oocytes cultured for 44 h *in vitro*, and supplementation with 100 μM AAI had a significant reduction compared to the controls (control: 78.3 ± 2.3%, n = 183; 10 μM: 78.7 ± 1.5%, n = 192; 25 μM: 65.9 ± 1.7%, n = 190, P < 0.05; 50 μM: 53.1 ± 4.0%, n = 174, P < 0.01; 100 μM: 40.6 ± 2.1%, n = 186, P < 0.001; Fig. 1B). Thus, treatment of 100 μM AAI was used for subsequent investigations. Collectively, these observations suggest that AAI exposure leads to the failure of porcine oocyte meiotic progression.

### AAI exposure impairs the spindle assembly and chromosome alignment in porcine oocytes

**Figure 2:**
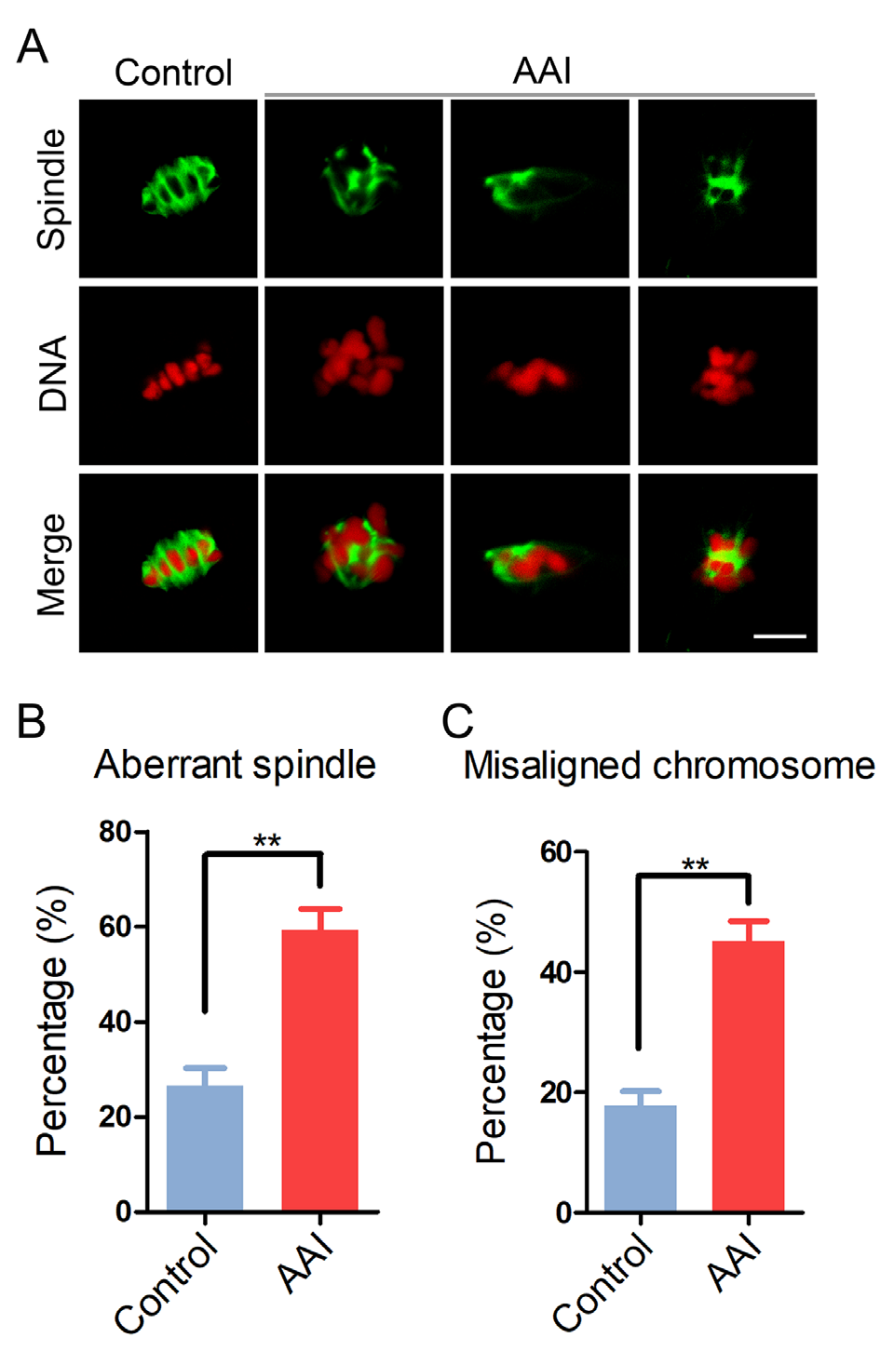
**Effects of AAI exposure on the spindle/chromosome structure in porcine oocytes.** (**A**) Representative images of spindle morphologies and chromosome alignment in control and AAI-exposed oocytes. Oocytes were immunostained with anti–α-tubulin-FITC antibody to visualize the spindles and were counterstained with propidium iodide (PI) to visualize the chromosomes. Scale bar, 10 μm. (**B**) The rate of aberrant spindles was recorded in control and AAI-exposed oocytes. (**C**) The rate of misaligned chromosomes was recorded in control and AAI-exposed oocytes. Data in (**B**) and (**C**) were presented as mean percentage (mean ± SEM) of at least three independent experiments. **P < 0.01.

Since the meiotic arrest of oocytes is highly correlated with the defective spindle assembly and chromosome alignment, we tested whether this is the case in AAI-exposed oocytes. To this end, M I oocytes were immunostained with anti-α-tubulin-FITC antibody to observe the spindle organization and counterstained with PI (Propidium Iodide) to visualize the chromosome alignment. The immunostaining results showed that a large number of control oocytes displayed a typical barrel-shape spindle apparatus with a well-aligned chromosome on the equatorial plate (spindle: 26.6 ± 3.5%, n = 90; chromosome: 17.8 ± 2.4%, n = 96; Fig. 2A, B, C). By striking contrast, a higher frequency of various morphology-aberrant spindles with misaligned chromosomes were present in AAI-exposed oocytes (spindle: 59.2 ± 4.5%, n = 91, P < 0.01; chromosome: 45.2 ± 3.3%, n = 91, P < 0.01; Fig. 2A, B, C), indicating that AAI exposure causes the spindle/chromosome defects.

### AAI exposure decreases the acetylation level of α-tubulin in porcine oocytes

**Figure 3:**
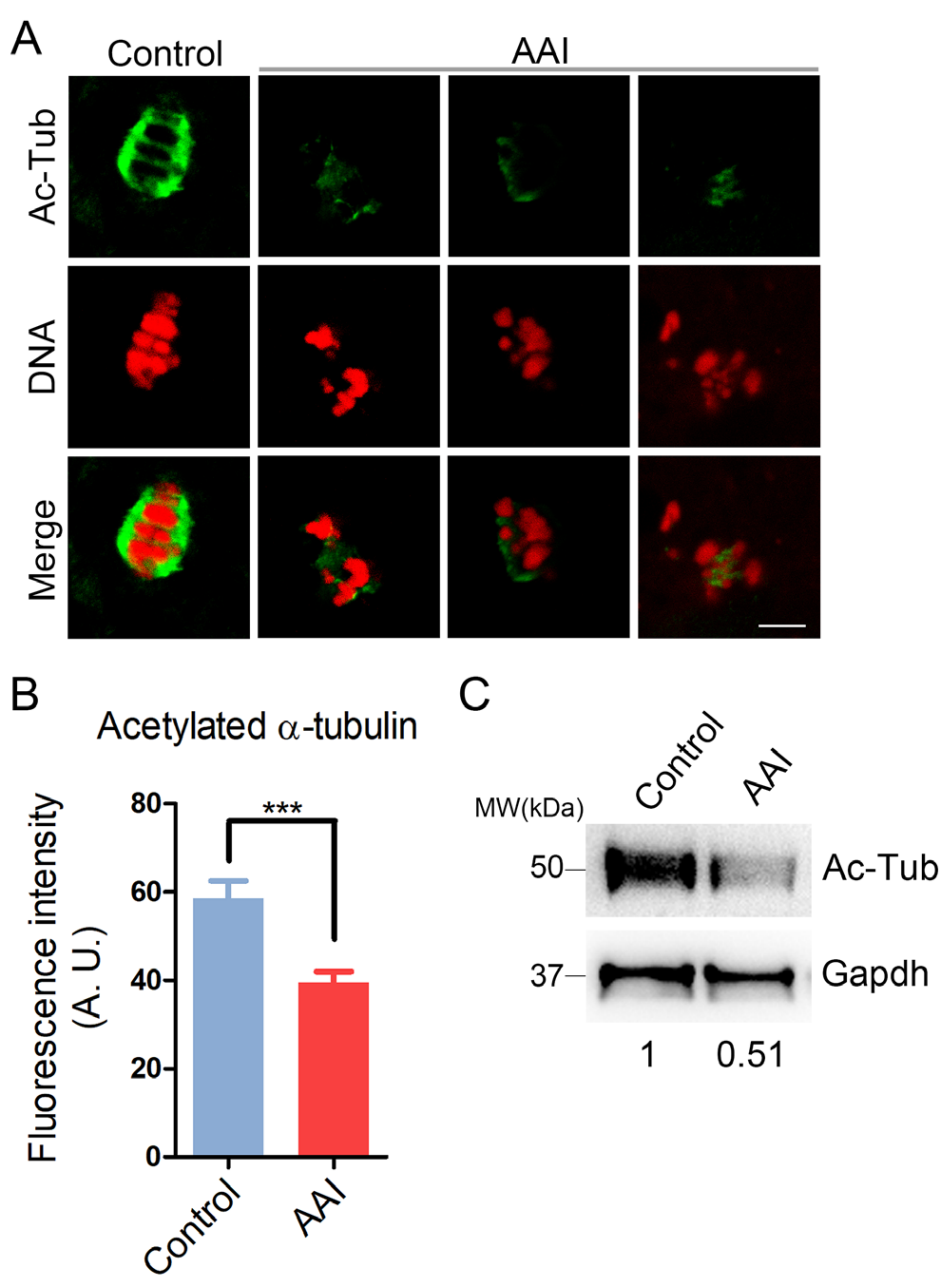
**Effects of AAI exposure on the acetylation level of α-tubulin in porcine oocytes.** (**A**) Representative images of acetylated α-tubulin (Ac-Tub) in control and AAI-exposed oocytes. Oocytes were immunostained with anti-acetyl-α-tubulin (Lys-40) antibody to assess the acetylation level of α-tubulin. Scale bar, 10 μm. (**B**) Quantitative measurement of the fluorescence intensity of acetylated α-tubulin in control and AAI-exposed oocytes. Data were presented as mean percentage (mean ± SEM) of at least three independent experiments. ***P < 0.001. (**C**) The acetylation levels of α-tubulin in control and AAI-exposed oocytes were examined by western blotting. The blots were probed with anti-acetyl-α-tubulin (Lys-40) antibody and anti-Gapdh antibody, respectively.

To ask whether the defects of spindle assembly were caused by the impaired microtubule dynamics in AAI-exposed oocytes, we assessed the level of acetylated α-tubulin, an indicator of the stabilized microtubules that has been previously proven in oocytes. We observed that AAI exposure considerably decreased the signals of acetylated α-tubulin compared to the controls as judged by the immunofluorescence analysis (39.5 ± 2.4, n = 34 vs 58.5 ± 4.0, n = 35, P < 0.001; Fig. 3A, B), which was further confirmed by the western blotting (Fig. 3C). This observation suggests that AAI exposure renders microtubules less stable and hence compromises the spindle assembly.

### AAI exposure disrupts actin cytoskeleton in porcine oocytes

**Figure 4:**
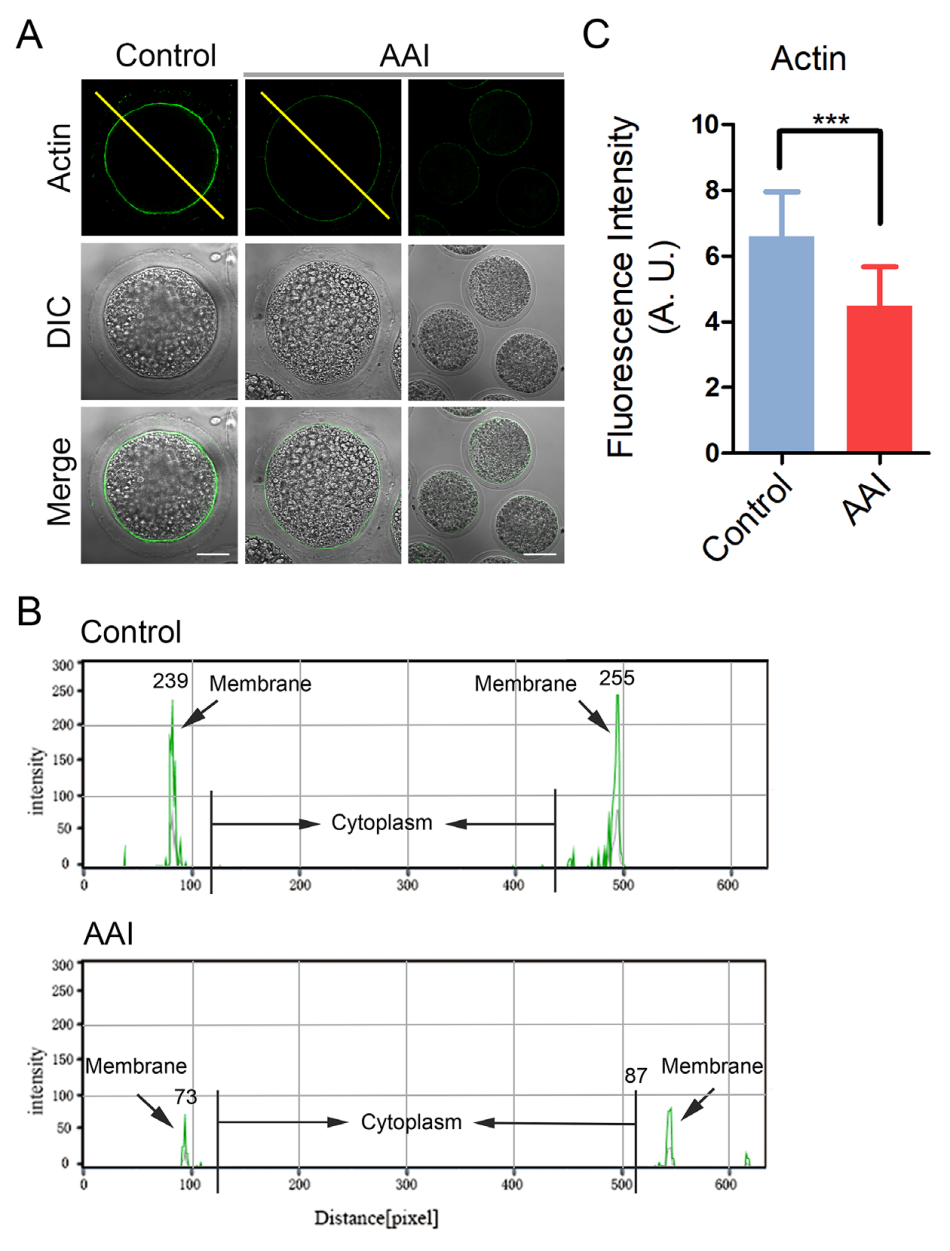
**Effects of AAI exposure on the actin dynamics in porcine oocytes.** (**A**) Representative images of actin filaments in control and AAI-exposed oocytes. Oocytes were immunostained with anti-phalloidin-FITC antibody to visualize the actin filaments. Scale bar, 25 and 60 μm. (**B**) The fluorescence intensity profiling of actin filaments in oocytes. Lines were drawn through the oocytes, and pixel intensities were quantified along the lines. (**C**) The fluorescence intensity of actin signals was measured in control and AAI-exposed oocytes. Data were presented as mean percentage (mean ± SEM) of at least three independent experiments. ***P < 0.001.

Actin cytoskeleton plays a critical role in the spindle positioning and cortical polarization during oocyte meiotic maturation. To determine whether actin dynamics is involved in the AAI exposure-induced meiotic failure, phalloidin-TRITC was applied to stain the F-actin. As shown in Fig. 4A, actin filaments were accumulated evenly on the plasma membrane with robust signals in control oocytes. However, AAI-exposed oocytes displayed a discontinuous distribution of actin with faded signals, which were assessed by the fluorescence plot profiling that were quantified along the lines drawn through the oocytes (Fig. 4A, B). The measurement of fluorescence intensity on the plasma membrane also indicated that actin signals substantially declined in the AAI-exposed oocytes compared to the controls (6.6 ± 0.2, n = 37 vs 4.5 ± 0.2, n = 37, P < 0.001; Fig. 4C), implying that AAI exposure disrupts the actin dynamics to impede the normal meiotic maturation of porcine oocytes.

### AAI exposure compromises the mitochondrial integrity in porcine oocytes

**Figure 5:**
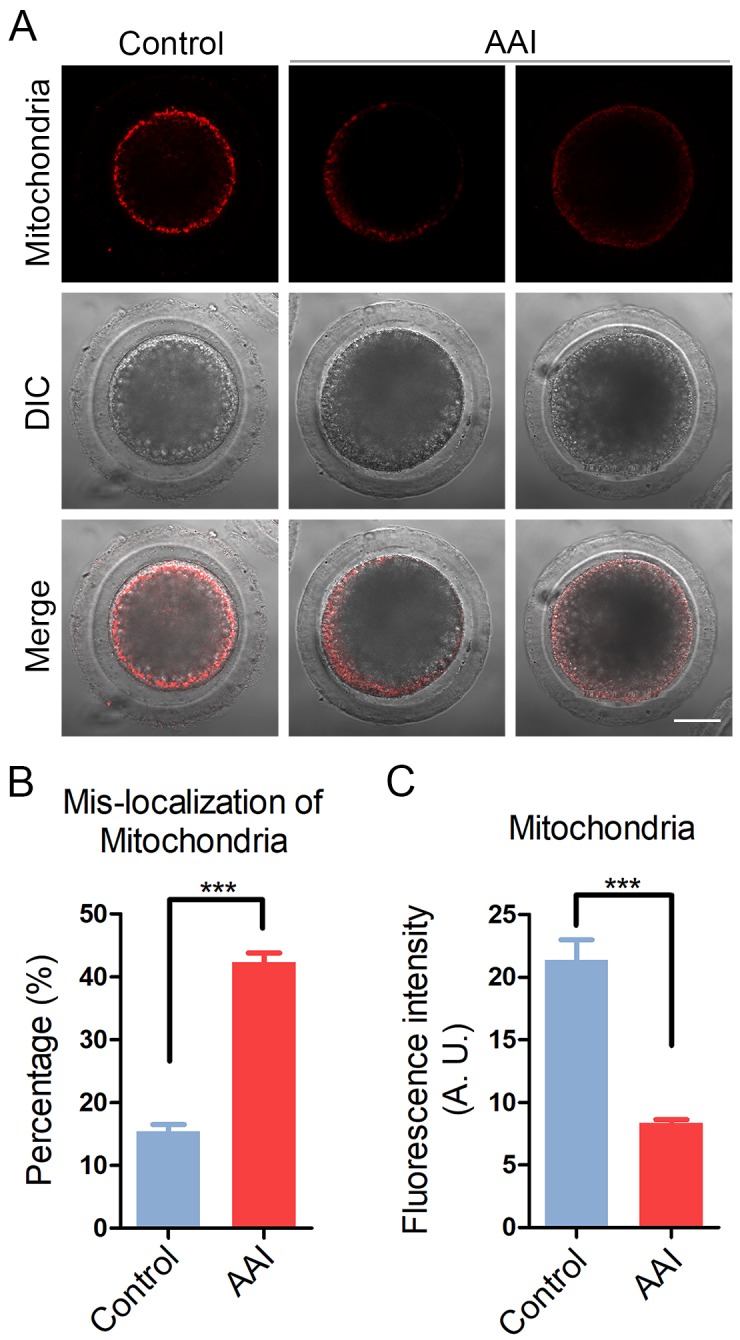
**Effects of AAI exposure on the distribution of mitochondria in porcine oocytes.** (**A**) Representative images of mitochondrial staining in control and AAI-exposed oocytes. Scale bar, 20 μm. (**B**) Abnormal rates of mitochondrial distribution in control and AAI-exposed oocytes. (**C**) The fluorescence intensity of mitochondrial signals was measured in control and AAI-exposed oocytes. Data in (**B**) and (**C**) were presented as mean percentage (mean ± SEM) of at least three independent experiments. ***P < 0.001.

Mitochondrial integrity is essential for oocyte development and considered as one of the key indicators of cytoplasmic maturation of oocytes, we then observed its changes following AAI exposure. By MitoTrackerR staining, we found that in control oocytes most of mitochondria distributed around the plasma membrane (Fig. 5A). By contrast, in AAI-exposed oocytes mitochondria entirely or partially lost their normal localization with much weaker signals (Fig. 5A). The mis-localization rate was increased from 15% in controls to 42% in AAI-exposed oocytes (15.4 ± 1.1%, n = 92 vs 42.3 ± 1.5%, n = 95, P < 0.001; Fig. 5B). Furthermore, the measurement of fluorescence intensity showed an obvious decline of mitochondria signals in AAI-exposed oocytes compared to the controls (21.4 ± 1.6, n = 35 vs 8.3 ± 0.3, n = 35, P < 0.001; Fig. 5C), indicating that the distribution and function of mitochondria was damaged.

### AAI exposure disrupts the proper localization of cortical granules and ovastacin in porcine oocytes

**Figure 6:**
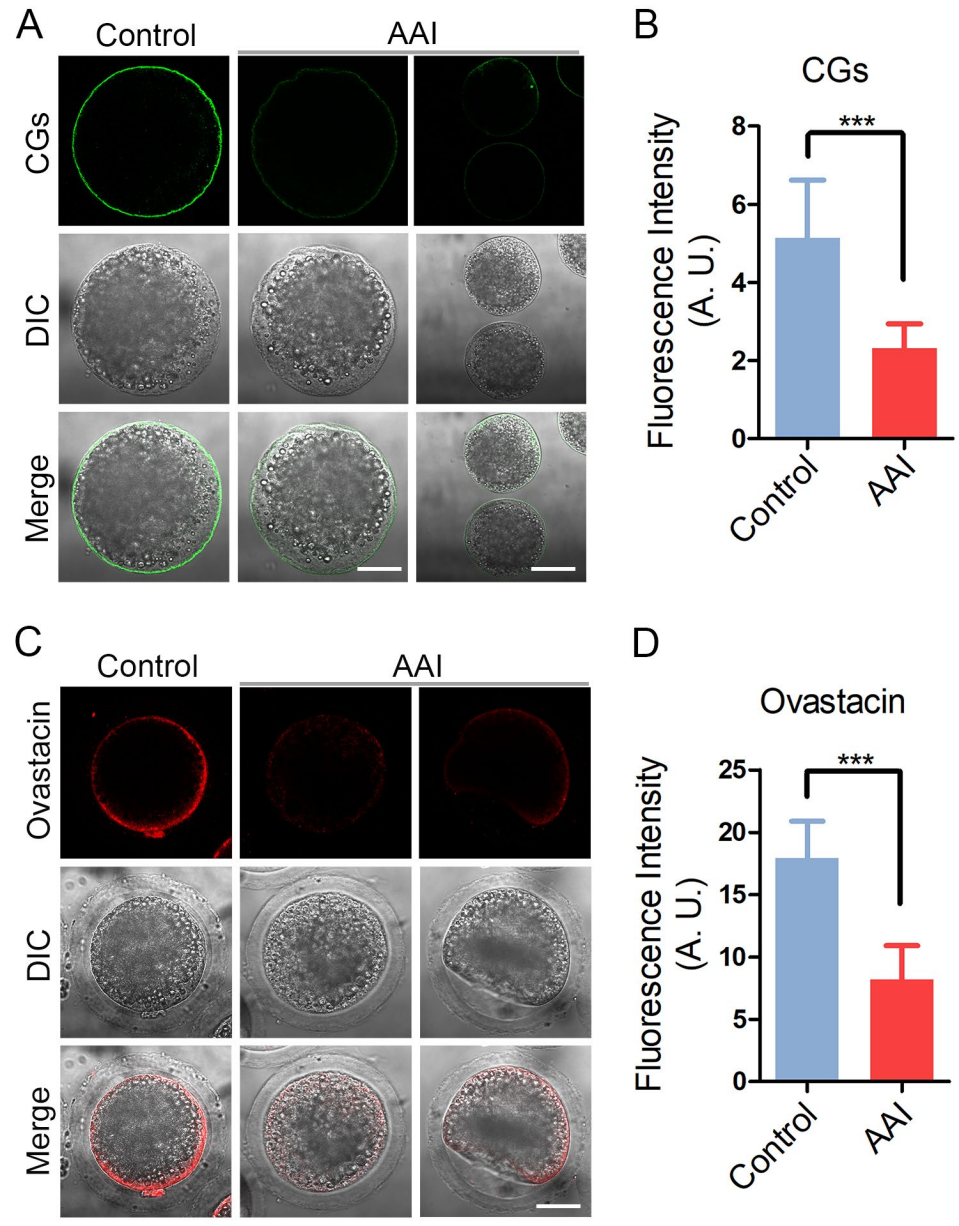
**Effects of AAI exposure on the distribution of cortical granules and ovastacin in porcine oocytes.** (**A**) Representative images of cortical granule localization in control and AAI-exposed oocytes. MII oocytes cultured for 44 h *in vitro* were stained with LCA-FITC to display the cortical granules. Scale bar, 30 and 60 μm. (**B**) The fluorescence intensity of cortical granules was measured around the signals on the plasma membrane in control and AAI-exposed oocytes. (**C**) Representative images of ovastacin localization in control and AAI-exposed oocytes. Ovastacin was immunostained with rabbit polyclonal anti-human ovastacin antibody and imaged by confocal microscope. Scale bar, 30 μm. (**D**) The fluorescence intensity of ovastacin was measured in control and AAI-exposed oocytes. Data in (**B**) and (**D**) were presented as mean percentage (mean ± SEM) of at least three independent experiments. ***P < 0.001.

CGs (cortical granules) are oocyte-specific vesicles that are exocytosed to the extracellular space of oocytes following fertilization to function in the block to polyspermy. It is noteworthy that proper distribution of CGs is usually regarded as another important indicator of oocyte cytoplasmic maturation. Therefore, we examined whether AAI exposure would influence the distribution of CGs using their marker LCA-FITC. The immunostaining results revealed that CGs located under the subcortical region of oocytes with even and strong signals (Fig. 6A). However, they lost the normal localization in the subcortex in AAI-exposed oocytes by displaying the inconsecutive and much weaker signals (Fig. 6A). The quantitative analysis of fluorescence intensity indicated that CG signals had a remarkable reduction in AAI-exposed oocytes in comparison with the controls (5.1 ± 0.3, n = 33 vs 2.3 ± 0.1, n = 34, P < 0.001; Fig. 6B).

In addition, we also examined the distribution of ovastacin, a first identified component of CGs in mammals that is required for the post-fertilization cleavage of sperm binding site in the zona pellucida to prevent polyspermy. Consistent with the above observation, abnormal distribution of ovastacin was present in AAI-exposed oocytes by showing the loss of even and continuous localization and much more decreased intensity of fluorescence than that in controls (17.9 ± 0.5, n = 31 vs 8.2 ± 0.5, n = 33, P < 0.001; Fig. 6C, D), implicating that sperm binding site might be prematurely lost in AAI-exposed unfertilized oocytes.

### AAI exposure weakens the sperm binding ability and fertilization potential of porcine oocytes

**Figure 7:**
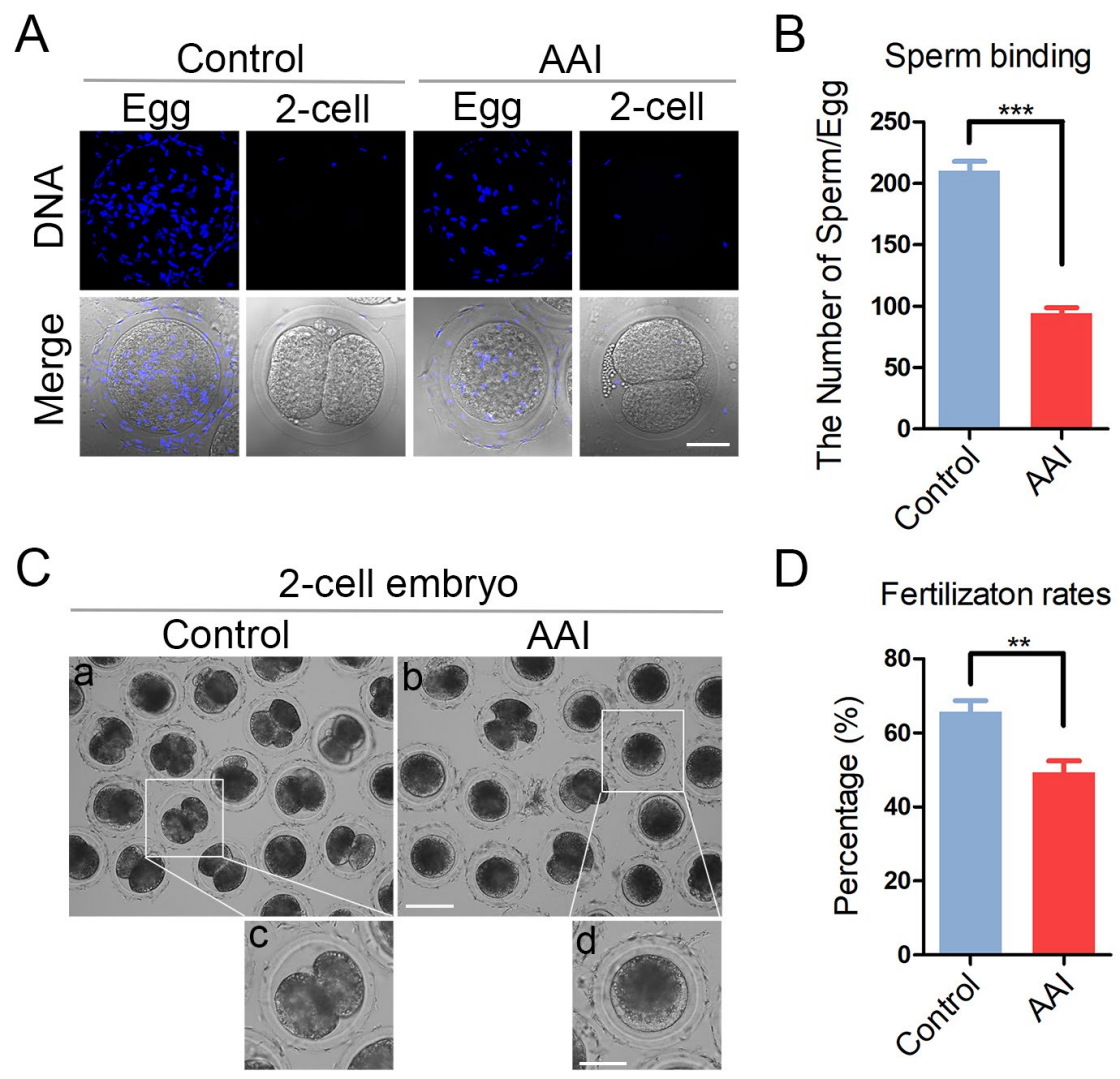
**Effects of AAI exposure on the sperm binding and fertilization of porcine oocytes.** (**A**) Representative images of eggs and two-cell embryos bound by sperm. Eggs and two-cell embryos from control and AAI-exposed groups were incubated with capacitated sperm for 1 h to carry out the sperm binding assay. Scale bar, 30 μm. (**B**) The number of sperm binding to the surface of zona pellucida surrounding eggs from control and AAI-exposed groups was counted, respectively. (**C**) Representative images of fertilized eggs in control and AAI-exposed groups. Scale bar, 100μm (a, b); 50 μm (c, d). (**D**) *In vitro* fertilization rate was recorded in control and AAI-exposed oocytes. Data in (**B**) and (**D**) were presented as mean percentage (mean ± SEM) of at least three independent experiments. **P < 0.01, ***P < 0.001.

To examine whether abnormal distribution of ovastacin would result in the sperm binding defect in AAI-exposed oocytes, sperm-zona binding assay was carried out. The sperm head was counterstained with Hoechst to count the number of sperm binding to the zona pellucida surrounding unfertilized eggs, and two-cell embryos were used as negative control. In control unfertilized eggs, we observed that zona pellucida was able to robustly support the binding of a large number of sperm, conversely in control two-cell embryos, zona pellucida no longer supported any sperm binding due to the loss of sperm binding site following fertilization (Fig. 7A). Whereas, in AAI-exposed unfertilized eggs, the number of sperm binding to the zona pellucida was significantly reduced in comparison with the controls (210.0 ± 7.8, n = 46 vs 94.3 ± 4.5, n = 44, P < 0.001; Fig. 7A, B).

Then we further tested the fertilization potential of AAI-exposed oocytes. We found that a large proportion of control oocytes were able to be fertilized and develop to two-cell embryos, while AAI-exposed oocytes showed a remarkably reduced fertilization rate (65.7 ± 3.0%, n = 161 vs 49.2 ± 3.1%, n = 148, P < 0.01; Fig. 7C, D). Taken together, these observations suggest that AAI exposure leads to the impaired sperm binding ability of oocytes which might be caused by the precocious release of ovastacin, and thereby weakening the fertilization potential.

### AAI exposure elevates the levels of ROS and DNA damage in porcine oocytes

**Figure 8:**
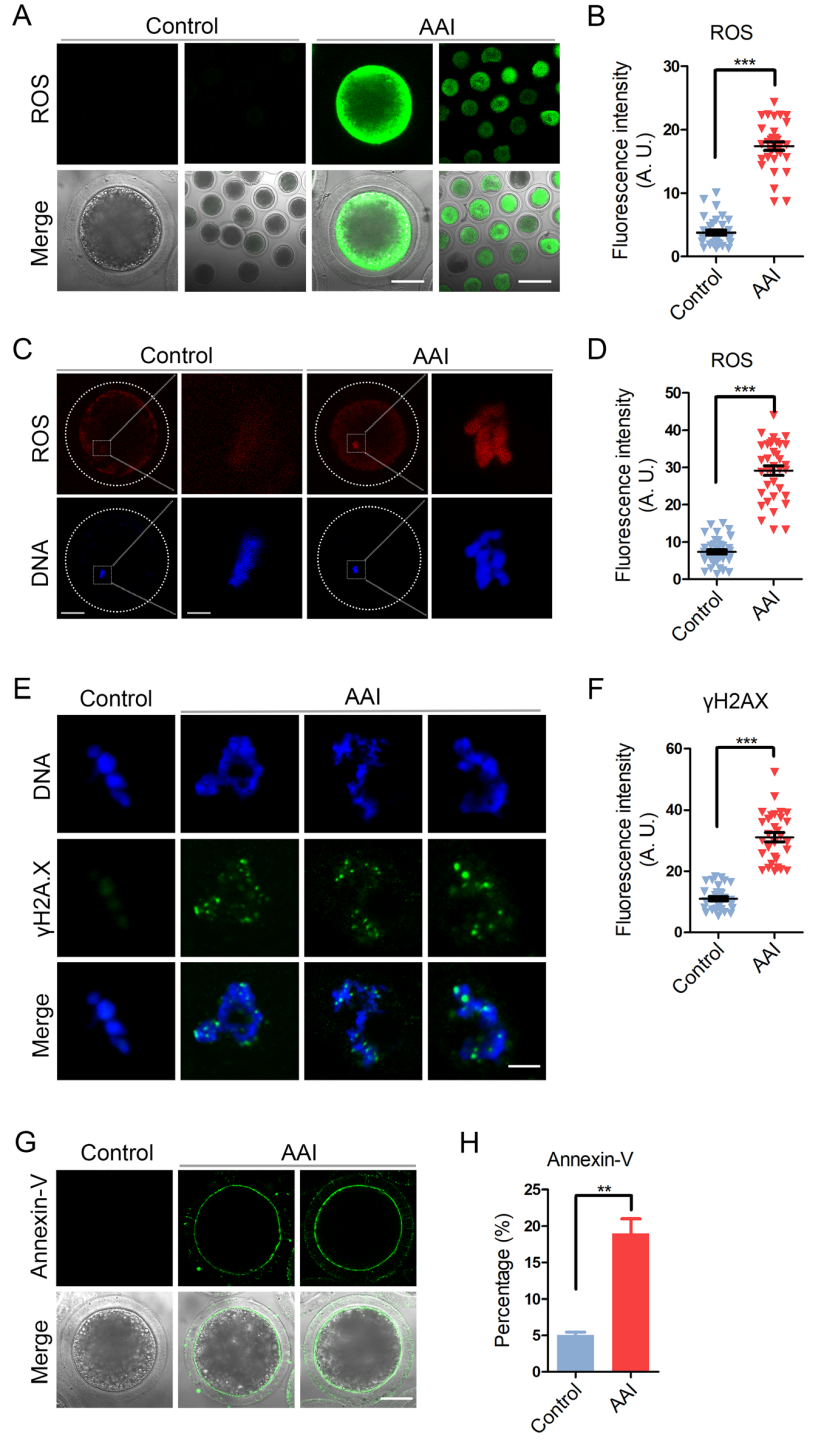
**Effects of AAI exposure on the ROS level, DNA damage and early apoptosis in porcine oocytes.** (**A**) Representative images of DCFH staining in control and AAI-exposed oocytes. Scale bar, 40 and 80 μm. (**B**) The fluorescence intensity of ROS levels was recorded in control and AAI-exposed oocytes. (**C**) Representative images of DHE staining in control and AAI-exposed oocytes. Scale bar, 20 and 5 μm. (**D**) The fluorescence intensity of ROS levels was recorded in control and AAI-exposed oocytes. (**E**) Representative images of DNA damage in control and AAI-exposed oocytes. Scale bar, 5 μm. (**F**) The fluorescence intensity of γH2AX signals was measured in control and AAI-exposed oocytes. (**G**) Representative images of apoptotic oocytes in control and AAI-exposed groups. Scale bar, 40 μm. (**H**) The rate of apoptotic oocytes was recorded in control and AAI-exposed groups. Data in (**B**), (**D**), (**F**) and (**H**) were presented as mean percentage (mean ± SEM) of at least three independent experiments. **P < 0.01, ***P < 0.001.

AAI has been reported to adversely induce the oxidative stress and increase the DNA damage level in various cells to impair the normal cellular functions, which speeds up the apoptotic progression. To confirm if this is the case during oocyte development, we examined them in AAI-exposed oocytes. As judged by both DCFH (dichloroflorescein) and DHE (dihydroethidium) staining as well as intensity quantification, AAI exposure apparently increased the ROS levels compared to the controls (DCFH: 3.8 ± 0.4, n = 33 vs 17.4 ± 0.7, n = 33, P < 0.001; DHE: 7.3 ± 0.6, n = 37 vs 29.2 ± 1.3, n = 36, P < 0.001; Fig. 8A, B, C, D). Also, the level of DNA damage was remarkably elevated in AAI-exposed oocytes in comparison with the controls (11.0 ± 0.7, n = 31 vs 31.1 ± 1.5, n = 30, P < 0.001; Fig. 8E, F).

Next, we examined the apoptosis status of oocytes by Annexin-V staining to detect the translocation of phosphatidylserine from the inner to the outer leaflet of the oocyte membrane. The results showed that green fluorescent signals were hardly detected in control oocytes, but clearly found on the membrane in AAI-exposed oocytes, indicative of early apoptosis (Fig. 8G). The rate of apoptotic rate was dramatically higher in AAI-exposed group than that in controls (5.1 ± 0.4%, n = 97 vs 19.0 ± 2.0%, n = 89, P < 0.01; Fig. 8H).

## DISCUSSION

Since 1994, increasing countries have banned or issued a consumer advisory against the botanical products containing AA, including dietary supplements [17]. Nonetheless, AA can still be detected in herbal medicine and diet pills at concentrations from 2.1 ppm (6.1 μM) to 668 ppm (1964 μM) [18,19]. AA-containing Chinese herbs have been a worldwide issue because of their association with toxicity to multiple organs, including the stomach, intestine and liver [1,20,21]. They also induce tumors of various types and severe damage to ovarian development in mammals [15,22,23]. However, the effect of AA exposure on the oocyte quality has not been investigated yet.

To explore the potential impact of AAI exposure on the oocyte meiotic progression, we selected pig oocytes as a model because they share the physiological and developmental similarities with human oocytes compared with mouse oocytes, which could provide a more accurate data for the human reproductive research. We first examined the effect of AAI exposure on the first polar body extrusion, a critical developmental indicator for the oocyte meiotic progression. The findings showed that AAI-exposed oocytes displayed a significantly decreased rate of polar body extrusion with poor expansion of cumulus cells, suggesting that AAI exposure perturbs the normal oocyte meiotic progression. This was further evidenced by the observations that a higher frequency of disrupted spindles and misaligned chromosomes was present in the AAI-exposed oocytes. Additionally, our data validated that AAI exposure remarkably decreased the acetylation level of α-tubulin, an indicator of stable microtubules, indicating that the loss of microtubule stability might be one of the leading causes resulting in the defective spindle assembly.

Another cytoskeletal structure actin is known to play an important part in the intracellular transport, spindle positioning and meiotic progression in oocytes [24]. Our findings illustrated that the impaired actin dynamics might be another reason leading to the oocyte meiotic arrest when exposed to AAI.

During oocyte meiotic maturation, mitochondrial integrity has been considered as one of the indicators of cytoplasmic maturation. In addition, mitochondrion is the primary organelle that generates ATP for normal oocyte development [25]. We found the aberrant distribution pattern and reduced amount of mitochondria in AAI-exposed oocytes, suggesting that AAI exposure causes the mitochondrial dysfunction and compromised cytoplasmic maturation in oocytes.

CGs form a uniform layer in the subcortical region of fully grown oocytes to function in the post-fertilization block to polyspermy [26]. Our data showed that the amount of CGs was substantially decreased in the subcortex of AAI-exposed oocytes, confirming that cytoplasmic maturation is disrupted as a result of AAI exposure. Consistently, the distribution and the amount of ovastacin, a component of cortical granules, which cleaves ZP2 at the N-terminus following fertilization to definitively block the sperm binding to the zona pellucida [26], was also perturbed in AAI-exposed oocytes. This was in line with the observation that much less sperm bound to the zona pellucida surrounding AAI-exposed oocytes, consequently leading to the decline of fertilization rate.

Finally, our findings revealed that AAI-exposed oocytes displayed dramatically higher levels of ROS, DNA damage and apoptosis, which might be cause for the AAI exposure-induced defects of oocyte quality and fertilization ability.

In conclusion, our study provides a body of evidence documenting that AAI exposure impairs the oocyte meiotic progression and fertilization capacity via compromising both nuclear maturation and cytoplasmic maturation, which might be meditated by the excessive oxidative stress-induced DNA damage and apoptosis. Therefore, exposure to AAI not only does harm to somatic cells which may lead to various cancers, but also compromises the quality of germ cells, which might increase the possibility to suffer from the subfertility or infertility in humans and animals.

## MATERIALS AND METHODS

### Antibodies

Mouse monoclonal anti-α-tubulin FITC antibody, anti-phalloidin-TRITC antibody, anti-acetyl-α-tubulin (Lys-40) antibody and lens culinaris agglu-tinin (LCA)-FITC were purchased from Sigma (St. Louis, MO, USA); rabbit polyclonal anti-human ovastacin antibody was obtained from Dr. Jurrien Dean (NIH); FITC-conjugated goat anti-mouse IgG (H + L) and TRITC-conjugated goat anti-mouse IgG (H + L) were purchased from Zhongshan Golden Bridge Biotechnology Co., LTD (Beijing, China).

### Porcine oocyte collection and *in vitro* maturation

For *in vitro* maturation (IVM), ovaries were collected from prepubertal gilts at a local abattoir and transported to the laboratory in a 0.9% NaCl solution containing penicillin G (75 mg/ml) and streptomycin sulphate (50 mg/ml). Soon afterwards ovaries were washed twice with sterile phosphate-buffered saline (PBS), the Cumulus-oocyte complexes (COCs) were subsequently aspirated from medium-sized follicles (3-6 mm in diameter) using a 20-gauge needle attached to a 20 ml disposable syringe. COCs surrounded by a compact cumulus mass with evenly granulated cytoplasm were washed three times with maturation medium, separated from the cellular debris, and then transferred to the maturation medium. The basic maturation medium was improved TCM-199 supplemented with 75 μg/ml of penicillin, 50 μg/ml of streptomycin, 0.5 μg/ml of LH, 0.5 μg/ml of FSH, 10 ng/ml of EGF and 0.57 mM cysteine. To prepare mature oocytes *in vitro*, a group of 30 COCs was transferred to 100 μl of maturation medium and then covered with 100 μl paraffin oil to culture at 38.5°C in a humidified atmosphere of 5% CO_2_.

### AAI treatment

AAI was purchased from Macklin Biochemical Co., Ltd (Shanghai, China). AAI was dissolved in dimethylsulphoxide (DMSO) and diluted into a final concentration of 10, 25, 50 and 100 μM, respectively, with maturation medium, with the final concentration of the solvent not more than 0.1% of the culture medium.

### Immunofluorescence and confocal microscopy

Denuded oocytes (DOs) were fixed in 4% paraformaldehyde (PFA) in PBS for 1 h at room temperature. Oocytes were washed three times in PBS, and then rehydrated and transferred to the permeabilization solution (1% Triton X-100, 20 mM HEPES, pH 7.4, 3 mM MgCl_2_, 50 mM NaCl, 300 mM sucrose, 0.02% NaN_3_ in PBS) for 8-12 h. After blocking with 3% BSA for 1 h at room temperature, oocytes were incubated with anti-α-tubulin-FITC antibody (1:200), anti-acetylated tubulin antibody (1:100), phalloidin-FITC (1:200) or anti-ovastacin antibody (1:100) at 4°C overnight, followed by incubation with an appropriate secondary antibody for 1 h and counterstaining of PI (propidium iodide) for 10 min at room temperature. Finally, oocytes were mounted on glass slides and observed under a laser-scanning confocal fluorescent microscope (Zeiss LSM 700 META).

For the measurement of fluorescence intensity, signals from both control and treatment oocytes were acquired by performing the same immunostaining procedure and setting up the same parameters (pinhole: 1AU; gain: 1000) as those used with the confocal microscope. ImageJ (NIH, Bethesda, MD, USA) was used to define a region of interest (ROI), and the average fluorescence intensity per-unit area within the ROI was determined. Independent measurements that used identically sized ROIs were taken for the cell membrane and cytoplasm. The average values of all measurements were used to compare the final average intensities between the control and treatment groups.

### Evaluation of mitochondrion distribution

COCs at MII stage following IVM for 44 h were placed to the maturation medium containing 2 mg/ml hyaluronidase to remove the cumulus cells. DOs were stained for active mitochondria in the maturation medium containing 500nM cell permeant MitoTracker Red CMXRos (ThermoFisher) for 30 min at 38.5°C in a dark environment and 5% CO2 in air. After washing three times with maturation medium for 20 min each, oocytes were mounted in DPBS containing 0.1% BSA (pH 7.4) on the non-fluorescent glass slides and observed under the laser-scanning confocal microscope at room temperature.

### Western blotting analysis

A total of 100 porcine oocytes was collected and lysed in 4× NuPAGE™ LDS sample buffer (ThermoFisher, USA) containing protease inhibitor, and then separated on 10% Bis-Tris precast gels and transferred onto polyvinylidene difluoride (PVDF) membranes. The blots were blocked in TBST containing 5% low fat dry milk for 1 h at room temperature and then incubated with anti-acetylated tubulin antibody (1:1000) or anti-Gapdh (1:5000) antibody overnight at 4°C. After washing in TBST, the blots were incubated with horseradish peroxidase (HRP)-conjugated secondary antibodies for 1 h at room temperature. Chemiluminescence was detected with ECL Plus (GE Healthcare, USA) and protein bands were acquired by Tanon-3900 (Tanon, China).

### Sperm binding assay

A 0.1 ml frozen semen pellet was thawed at 38.5°C in 10 ml of sperm-washing medium. After washing twice by centrifugation (1,900 × g at room temperature, 4 min), the ejaculated cryopreserved spermatozoa were resuspended in the fertilization medium to a concentration of 1 × 10^6^ cells/ml and capacitated by an additional 1 h of incubation at 38.5°C. 50 μl of the sperm sample was added to the fertilization droplets containing the oocytes, giving a final sperm concentration of 0.25 × 10^6^ cells/ml. Then oocytes were co-incubated with sperm for 1 h. Sperm binding to matured oocytes or two-cell embryos from control and AAI-exposed groups were observed using the control two-cell embryos as a negative wash control. Samples were fixed in 4% PFA for 30 min, counterstained with Hoechst 33342. Bound sperm were quantified from z projections acquired by the confocal microscope, and results reflect the mean ± SEM from at least three independently obtained samples, each containing 10-12 porcine oocytes/embryos.

### *In vitro* fertilization

Matured oocytes with a first polar body were washed 3 times in modified Tris-buffered medium. Approximately 30 to 35 oocytes were transferred into a 50 μl droplet of IVF medium covered with the mineral oil that had been equilibrated at 38.5°C in 5% CO_2_ in air. A 0.1 ml frozen semen pellet was thawed at 38.5°C in 10 ml of sperm-washing medium. After washing twice by centrifugation (1,900 × g at room temperature, 4 min), the ejaculated cryopreserved spermatozoa were resuspended in the fertilization medium to a concentration of 1 × 10^6^ cells/ml and capacitated by an additional 1 h of incubation at 38.5°C. 50 μl of sperm sample was added to the fertilization droplets containing 30 to 35 matured oocytes, giving a final sperm concentration of 0.25 × 10^6^ cells/ml, and then co-incubated for 6 h. After fertilization, oocytes were washed 3 times and cultured with 500 μl of porcine zygote medium in 4-well dishes at 38.5°C, 5% CO_2_. Cleavage formation was evaluated on day 2 after IVF and designated as the successful fertilization.

### Determination of ROS generation

To determine the levels of intracellular ROS production, denuded oocytes by hyaluronidase treatment were incubated with the oxidation-sensitive florescent probe dichloroflorescein (DCFH, Beyotime Institute of Biotechnology, China) or dihydroethidium (DHE, Genmed Co. Ltd., China) for 30 min at 38.5°C. Then oocytes were washed three times in DPBS containing 0.1% BSA (pH 7.4) and mounted on the non-fluorescent glass slides. Fluorescent images were acquired by the confocal microscope at room temperature.

### Annexin-V staining

Denuded oocytes by hyaluronidase treatment were stained with the Annexin-V staining kit (Vazyme, Nanjing, China) according to the manufacturer’s instruction. After washing twice in DPBS, the viable oocytes were stained for 30 min in the dark with 90µl of binding buffer containing 10µl of Annexin-V-FITC. Then oocytes were washed three times in DPBS containing 0.1% BSA (pH 7.4) and mounted on the non-fluorescent glass slides. Fluorescent images were acquired by the confocal microscope at room temperature.

### Statistical analysis

All percentages from at least three repeated experiments were expressed as mean ± SEM, and the number of oocytes observed was labeled in parenthesesas (n). Data were analyzed by one-way ANOVA or *t* test, which was provided by SPSS 16.0 statistical software (SPSS, Chicago, IL, USA). The level of significance was accepted as *P < 0.05*.
